# Psychometric performance of EQ-5D-5L and SF-6DV2 in measuring health status of populations in Chinese university staff and students

**DOI:** 10.1186/s12889-023-17208-z

**Published:** 2023-11-22

**Authors:** Hui Jun Zhou, Aixue Zhang, Jie Wei, Jing Wu, Nan Luo, Pei Wang

**Affiliations:** 1 Business School, University of Shanghai for Science and Technology, Shanghai, China; 2 School of Public Health, Fudan University, 130 Dong An Road, Shanghai, 200032 China; 3 Key Lab of Health Technology Assessment, National Health Commission of the People’s Republic of China (Fudan University), Shanghai, China; 4 Department of TCM Manipulative Orthopedics, PLA Air Force Medical Center, Beijing, China; 5 School of Pharmaceutical Science and Technology, Tianjin University, Tianjin, China; 6 Saw Swee Hock School of Public Health, National University of Singapore, Singapore, Singapore

**Keywords:** EQ-5D-5L, SF-6DV2, Measurement properties, Utility, Health-related quality of life

## Abstract

**Aims:**

To compare measurement properties of EQ-5D-5L and SF-6DV2 in university staff and students in China.

**Methods:**

A total of 291 staff and 183 undergraduates or postgraduates completed the two instruments assigned in a random order. The health utility scores (HUS) of EQ-5D-5L and SF-6DV2 were calculated using the respective value sets for Chinese populations. The agreement of HUSs was examined using intraclass correlation coefficients (ICC) and Bland-Altman plot. Convergent validity of their HUSs and similar dimensions were assessed using Spearman’s correlation coefficient. Known-group validity of the HUSs and EQ-VAS score was assessed by comparing the scores of participants with and without three conditions (i.e., disease, symptom or discomfort, and injury), as well as number of any of the three conditions; their sensitivity was also compared.

**Results:**

The ICCs between the two HUSs were 0.567 (staff) and 0.553 (students). Bland-Altman plot found that EQ-5D-5L HUSs were generally higher. Strong correlation was detected for two similar dimensions (*pain/discomfort *of EQ-5D-5L and *pain *of SF-6DV2; *anxiety/depression *of EQ-5D-5L and *mental health *of SF-6DV2) in both samples. The correlation between the two HUSs were strong (0.692 for staff and 0.703 for students), and were stronger than their correlations with EQ-VAS score. All the three scores could discriminate the difference in three known-groups (disease, symptom or discomfort, number of any of the three conditions). The two HUSs were more sensitive than EQ-VAS score; and either of them was not superior than the other.

**Conclusions:**

Both EQ-5D-5L and SF-6DV2 HUSs have acceptable measurement properties (convergent validity, known-groups validity, sensitivity) in Chinese university staff and students. Nevetheless, only EQ-5D-5L (PD and AD) and SF-6DV2 (PN and MH) showed indicated good convergent validity as expected. Two types of HUSs cannot be used interchangeably, and each has its own advantages in sensitivity.

**Supplementary Information:**

The online version contains supplementary material available at10.1186/s12889-023-17208-z.

## Introduction

Generic preference-based measures EQ-5D and SF-6D are two well-known and widely used instruments to measure health-related quality of life (HRQOL), which can be converted into health utility scores (HUS), in clinical trial, economic evaluation or population health survey [1–4]. For example, EQ-5D was the HRQOL instrument in the China National Health Service Survey launched since 2008 [2]. EQ-5D and SF-6D both were recommended instruments for utility measurement for health economic evaluation by China Pharmaceutical Economics Evaluation Guidelines (2020 edition) [3].

The EQ-5D, which was developed by EuroQol in 1996 [5]. It currently has two versions, EQ-5D-3L and EQ-5D-5L, both of which include five dimensions.The original version EQ-5D-3L, categorizes the five dimensions into three severity levels: no problems, moderate problems, and extreme problems, capable of defining 243 (3^5^) unique health states. Because the EQ-5D-3L was found to be insensitive to mild or even moderate differences in HRQOL and greatly limited by the ceiling effect [6], EQ-5D-5L was developed in 2009 with two more levels in each dimension (slight problems and severe problems) to categorize health status [7]. The EQ-5D health states can be converted into HUS, which anchors at 1 (full health) and 0 (death), following country or population specific value sets. As expected, EQ-5D-5L has demonstrated better measurement properties than EQ-5D-3L [7–9]. The SF-6D, which was based on the SF-36 was developed by Brazier et al. in 2002 [10]. The SF-6D has two versions (SF-6DV1 & SF-6DV2) corresponding to the two versions of SF-36 [11]. SF-6DV1 has the disadvantages of unclear severity ordering of dimensions and limited sensitivity [12]. SF-6DV2 addresses them by simplifying level descriptions and providing clearer wording, and is thus with better reliability and validity [11, 13–15]. Similarly, each SF-6D health state can be translated into a HUS based on a certain value set for SF-6D.

Although both EQ-5D and SF-6D measure the same concept of HRQOL and provide HUS, their measurement performance was not the same in different populations, such as general populations in Asia [16–22]. For example, a study in China general population suggested that SF-6DV2 is more sensitive in distinguishing participants with and without chronic diseases [22]; while a study in Thailand general population found a better sensitivity of EQ-5D-5L in distinguishing participants with different in characteristics gender, age, education level, household income, and number of diseases [17]. The studies generally not mentioned the use order of the two instruments, which is an important factor influencing the comparison results. In addition, no study has compared their performance in university staff and students.

Recently, an increasing number of studies have begun to measure the Health-Related Quality of Life (HRQOL) of university staff and students. Both populations are under great pressure of occupational overload and employment-related school performance, respectively [23–26]. This would adversely affect their physical and mental health and consequently HRQOL [23, 25]. Indeed, a study has shown that university staff have lower HRQOL than the general population in China [24]. However, as there are currently no specific measurement tools developed for assessing the HRQOL of university staff or students, the EQ-5D or SF-6D are commonly employed for this purpose [27–30]. Hence, it is important to select the most appropriate measurement tool based on different populations in use. This study thus aimed to compare the measurement properties between EQ-5D-5L and SF-6DV2 in university staff and students in China by randomly assigning their use order.

## Methods

### Study design and population

This is a web-based health survey targeted at the highly-educated populations, i.e., university staff and students currently working or studying in one of public universities. The questionnaire was distributed through the largest online survey platform in China, Wen Juan Xing (Changsha Ranxing Information Technology Co.,Ltd., Hunan, China). Wen Juan Xing, equivalent to Qualtrics, Survey Monkey or Cloud Research, provides online questionnaire design and survey functions for the customers. The study took a snowballing sampling method with a convenient sample composed of colleagues, friends and acquaintances. Then the questionnaire was circulated via Wechat working groups, personal invitation and unofficial announcement by the existing respondents among study population. The participation was completely voluntary and incentives were not provided in any form. The study was approved by the IRB committee of the Air Force Medical Center in Beijing (KongTe: NO 2021-169-PJ01).

### Data collection

The online questionnaire collected variables about health determinants such as demographic (age, gender, height, weight), lifestyle or behavioral (smoking, drinking) and socioeconomic (education, marital status) factors. Additionally, the conditions (diseases, symptoms, discomforts) which can directly influence individual HRQOL were systematically collected. In answering the online questionnaire, either EQ-5D-5L or SF-6DV2 was randomly assigned first and then followed by the other. This is to eliminate the ordering-effect when measuring the same property with different instruments.

### EQ-5D-5L

EQ-5D-5L inquires an individual’s HRQOL on the day of survey using two parts: a health-state descriptive system and a visual analog scale (EQ-VAS). The system includes five dimensions:*mobility (MO), self-care (SC), usual activities (UA), pain/discomfort (PD), and anxiety/depression (AD). *It measures 3125 health states in total, each expressed in five-digit numbers for EQ-5D-5L, combining the levels of five dimension each [31]. For example, EQ-5D-5L “52341” means extreme problems in mobility, slight problems in self-care, moderate problems in usual activities, severe pain/discomfort, and not anxious/depressed. In this study, we used the Chinese EQ-5D-5 L value set developed by Luo et al. to calculate EQ-5D-5L HUS [32] (Table1). The HUS for EQ-5D-5L state “52341” is 0.248. EQ-VAS is a 20 cm vertical visual scale, ranging from 0 (worst imaginable health) to 100 (best imaginable health), and reflecting the respondents’ self-rated overall health status [33].

**Table 1:** Characteristics of EQ-5D-5L and SF-6DV2

	EQ-5D-5L	SF-6DV2
Levels	5 levels	5–6 levels
Number of health states described	3,125	18,750
Formula of utility score calculation for China	1-(0.066*MO2 + 0.158*MO3 + 0.287*MO4 + 0.345*MO5 + 0.048*SC2 + 0.116*SC3 + 0.210*SC4 + 0.253*SC5 + 0.045*UA2 + 0.107*UA3 + 0.194*UA4 + 0.233*UA5 + 0.058*PD2 + 0.138*PD3 + 0.252*PD4 + 0.302*PD5 + 0.049*AD2 + 0.118*AD3 + 0.215*AD4 + 0.258*AD5)^a^	1-(0.038*PF2 + 0.008*PF3 + 0.140*PF4 + 0.395*PF5 + 0.050*RL2 + 0.059*RL3 + 0.096*RL4 + 0.097*RL5 + 0.047*SF2 + 0.060*SF3 + 0.093*SF4 + 0.108*SF5 + 0.047*PN2 + 0.083*PN3 + 0.154*PN4 + 0.388*PN5 + 0.427*PN6 + 0.033*MH2 + 0.050*MH3 + 0.132*MH4 + 0.134*MH5 + 0.029*VT2 + 0.060*VT3 + 0.108*VT4 + 0.116*VT5)^b^
The worst state value	“55555” -0.391	“555,655” -0.277

a Mobility (MO), Self-care (SC), Usual activities (UA), Pain/Discomfort (PD), Anxiety/Depression(AD),

b Physical functioning (PF), Role limitation (RL), Social functioning (SF), Pain (PN), Mental health (MH), and Vitality (VT)

### SF-6DV2

SF-6DV2 assess HRQOL of individuals covering the last 4 weeks in six dimensions i.e.*physical functioning (PF), role limitation (RL), social functioning (SF), pain (PN), mental health (MH), and vitality(VT)*, which have 5–6 functioning levels. SF-6DV2 can measure 18,750 health states, each of which is indicated by a six-digit number combining the levels in six dimensions. “312654” means your health limits you a little in moderate activities, you have no problems with your work or other regular daily activities as a result of your physical health or other activities as a result of your physical health or any emotional problems, your health limits your social activities a little of the time, you have pain extremely, you feel tense or downhearted and low all of the time, and you have a lot of energy a little of the time. The SF-6DV2 value set in China developed by Wu et al. was used in the study [15] (Table1). According to it, the HUS for SF-6DV2 state “312654” is 0.204.

### Statistical analysis

Descriptive statistic was conducted to depict respondent characteristics, the response distribution to the EQ-5D-5L and SF-6DV2 dimensions, their HUSs, EQ-VAS score, and the overall ceiling effects (the proportion of no problems in all the dimensions). Continuous variables were expressed as mean and standard deviation (SD), and categorical variables as frequency and percentage.

The agreement between EQ-5D-5L and SF-6DV2 HUSs was tested by intra-class correlation coefficient (ICC), which was computed with the two-way mixed effects model based on absolute agreement. ICC ranges from 0 to 1 and a value < 0.5, 0.5–0.75, and > 0.75 indicate excellent agreement poor, moderate, and good agreement, respectively [34–36]. Bland-Altman plots were also constructed to visually examine the utility differences of two instruments. The agreement is deemed perfect if the between-instrument differences have a mean of 0 and randomly scatter within the 1.96 SD around the mean [37, 38].

Convergent validity of EQ-5D-5L and SF-6DV2 similar dimensions (i.e., MO and SC vs. PF, UA and RL vs. SF, PD vs. PN, AD vs. MH) (Appendix 1) and their HUSs were evaluated by using the Spearman’s correlation coefficient (r): >0.5 (strong correlation), 0.35–0.5 (moderate correlation), 0.20–0.35 (weak correlation), and < 0.20 (poor correlation) [39].

Known-groups validity of EQ-5D-5L and SF-6DV2 HUSs was assessed by testing their ability in identifying different subgroups with known differences in health status. Following that, the sample have been classified independently according to the self-reported clinical conditions, i.e., disease, symptom or discomfort in 12 months, injury in 12 months, and number of the three conditions. Those with the condition or more conditions were believed to have worse health status than their respective counterparts. The p-value of the F test in ANOVA test was used as the indicator. Their sensitivity was compared using relative efficiency (RE), which was calculated based on the ratio of F-statistic values [40]. A higher RE indicates a better ability to detect statistically significant difference between subgroups. In this study, the F-statistic of EQ-5D-5L HUS was used as the reference to calculate the RE of SF-6DV2 HUS and EQ-VAS score. As a result, RE <1 means EQ-5D HUS is more effective.

Data were analyzed using SPSS 26.0 and STATA 17.0 software. All the analyses were two-sided and tested with a significance level of p < 0.05.

## Results

### Characteristics of the two samples

There were 474 respondents among which 291 were university staff. The student sample enrolled 99 undergraduates and 84 postgraduates (Table2). The mean ages of staff and students were 39 (9.6) years and 25.0 years (8.5) respectively. The faculty had slightly more males (55.3%) while student sample got more females (54.6%). The proportion of smoking habit was below 15% in both samples. The mean BMIs were 21.6(3.4) and 23.6(4.11) for staff and students respectively. A bigger proportion of students (54.6%) maintained normal BMI than faculty (54.6%). Compared to the students, staff reported higher prevalences of diseases and symptom/discomfort than the students but lower prevalence of injuries in the past year. These are expected as staff were older while students were more active and risk-taking.

**Table 2:** Characteristics of University staff and students (N = 474)

Characteristics	Options	University staff (n = 291)	University students (n = 183)
Gender	Male	161(55.3)	83(45.4)
	Female	130(44.7)	100(54.6)
Age (years)	< 18	0(0.0)	30(16.4)
	18–24	3(1.0)	75(41.0)
	25–40	162(55.7)	78(42.6)
	41–55	103(35.4)	0(0.0)
	> 55	23(7.9)	0(0.0)
Smoking	Smoke	41(14.1)	21(11.5)
	Never smoke	250(85.9)	162(88.5)
Drinking	Never drink	133(45.7)	83(45.4)
	One or fewer times per month	111(38.1)	79(43.2)
	More than twice a month	47(16.1)	21(11.4)
BMI	<18.5 (abnormal)	11(3.8)	21(11.5)
	18.5–24(normal)	159(54.6)	124(67.8)
	>24 (abnormal)	113(38.8)	38(20.8)
	Missing	8(2.7)	0(0.0)
Disease a	No	168(57.7)	159(86.9)
	Yes	122(42.0)	24(13.1)
	Missing	1(0.3)	0(0.0)
Symptom or discomfort in 12 months b	No	134(46.0)	114(62.3)
	Yes	156(53.7)	69(37.7)
	Missing	1(0.3)	0(0.0)
Injury in 12 months c	No	251(86.3)	129(70.5)
	Yes	33(11.3)	54(29.5.9)
	Missing	7(2.4)	0(0.0)

a Disease: hypertension, diabetes, kidney disease, heart disease, allergic diseases, other diseases

b Symptoms and discomfort: headache, back pain, pain in other areas; indigestion, nausea, sleep disturbance, emotional problems, and other discomforts

c Injury: Trauma (fracture, dislocated, muscle strain, cuts, abrasions, etc.), burns and scalds, and other conditions

### HRQOL profile

As shown in Table3, EQ-5D-5L was affected by the high ceiling effect that was 43.3% in measuring staff and 51.4% in measuring students. More than 92% of respondents reported “no problems” on “Mobility”, “Self-care” and “Usual activities” in both samples as they were generally considered healthy and able to carry out daily tasks. With regard to the PD and AD, 41.6% of staff and 30.1% of students reported problems on these two dimensions. The similar response distributions were observed in two samples while staff systematically reported more problems than students. Accordingly, the mean HUS of staff was 0.92 (0.11) which was lower than 0.95 (0.08) for students, and the mean VAS of staff was 77.5 (14.8) which was also lower than the students 84.5 (14.4).

**Table 3:** Distributions of responses to each of the EQ-5D-5L dimension in the two samples

Dimensions	Levels	University staff	University students
Mo a	Level 1	274(92.4)	176 (96.2)
	Level 2	13(4.5)	5 (2.7)
	Level 3	3(1.0)	2 (1.1)
	Level 4	1(0.3)	0(0.0)
	Level 5	0(0.0)	0(0.0)
SC b	Level 1	285(97.9)	182 (99.5)
	Level 2	2(0.7)	0(0.0)
	Level 3	3(1.0)	1 (0.5)
	Level 4	1(0.3)	0(0.0)
	Level 5	0(0.0)	0(0.0)
UA c	Level 1	279(95.9)	180(98.4)
	Level 2	8(2.7)	3(1.6)
	Level 3	2(0.7)	0(0.0)
	Level 4	2(0.7)	0(0.0)
	Level 5	0(0.0)	0(0.0)
PD d	Level 1	170(58.4)	128(69.9)
	Level 2	100(34.4)	52(28.4)
	Level 3	17(5.8)	3(1.6)
	Level 4	2(0.7)	0(0.0)
	Level 5	2(0.7)	0(0.0)
AD e	Level 1	163(56.0)	109(59.6)
	Level 2	102(35.1)	64(35.0)
	Level 3	20(6.9)	6(3.3)
	Level 4	4(1.4)	2(1.1)
	Level 5	2(0.7)	2(1.1)
Ceiling effect		126(43.3%)	94(51.4%)
Utility score	Mean(SD)	0.92(0.11)	0.95(0.08)
EQ-VAS	Mean(SD)	77.5(14.8)	84.5(14.4)
	Missing	9(3.0)	0(0.0)

a Mobility (MO); b Self-care (SC); c Usual activities (UA); d Pain/Discomfort (PD);

e Anxiety/Depression (AD)

The response distributions of SF-6DV2 exhibited a different pattern from EQ-5D-5L (Table4). Either university staff or students were found by SF-6DV2 to have more problems than by EQ-5D-5L. Less than half of staff sample reported “no problems” across each six dimensions. The worst was the VT dimension where only 30 (10.3%) faculty member did not feel tired in the past four weeks. Similar to the staff, students also had most problems in the VT dimension. Like EQ-5D-5L, students showed better HRQoL profile than staff. With more problems detected by SF-6DV2, the mean HUS in SF-6DV2 was 0.76 (0.14) in staff, which was 0.16 significantly lower than that derived by EQ-5D-5L. Likewise, the mean HUS of students was also significantly lower at 0.82 when measured by SF-6DV2 than EQ-5D-5L. The latter derived a mean HUS of 0.95 for students. The ceiling effects associated with the SF-6DV2 was lower than the EQ-5D-5L with 7.6% vs. 43.3% and 20.2% vs. 51.4% respectively in staff and students respectively.

**Table 4:** Distributions of responses to each of the SF-6DV2 dimension in the two samples

Dimensions	Levels	University staff	University students
PF a	Level 1	116(39.9)	126(68.9)
	Level 2	146(50.2)	50(27.3)
	Level 3	29(10.0)	3(1.6)
	Level 4	0(0.0)	3(1.6)
	Level 5	0(0.0)	1(0.5)
RL b	Level 1	84(28.9)	78(42.6)
	Level 2	83(28.5)	63(34.4)
	Level 3	112(38.5)	37(20.2)
	Level 4	10(3.4)	4(2.2)
	Level 5	2(0.7)	1(0.5)
SF c	Level 1	106(36.4)	99(54.1)
	Level 2	97(33.3)	56(30.6)
	Level 3	79(27.1)	24(13.1)
	Level 4	7(2.4)	4(2.2)
	Level 5	2(0.7)	0(0.0)
PN d	Level 1	134(46.0)	93(50.8)
	Level 2	69(23.7)	58(31.7)
	Level 3	70(24.1)	28(15.3)
	Level 4	15(5.2)	4(2.2)
	Level 5	2(0.7)	0(0.0)
	Level 6	1(0.3)	0(0.0)
MH e	Level 1	58(19.9)	62(33.9)
	Level 2	103(35.4)	60(32.8)
	Level 3	115(39.5)	49(26.8)
	Level 4	15(5.2)	12(6.6)
	Level 5	0(0.0)	0(0.0)
VT f	Level 1	30(10.3)	49(26.6)
	Level 2	69(23.7)	39(21.3)
	Level 3	140(48.1)	70(38.3)
	Level 4	42(14.4)	21(11.5)
	Level 5	9(3.0)	4(2.2)
	Missing	1(0.3)	0(0.0)
Ceiling effect		22(7.6%)	37(20.2%)
Utility scores	Mean (SD)	0.76(0.14)	0.82(0.14)

a Physical functioning (PF); b Role limitation (RL); c Social functioning (SF); d Pain (PN); e Mental health (MH); f Vitality (VT)

**Fig. 1:**
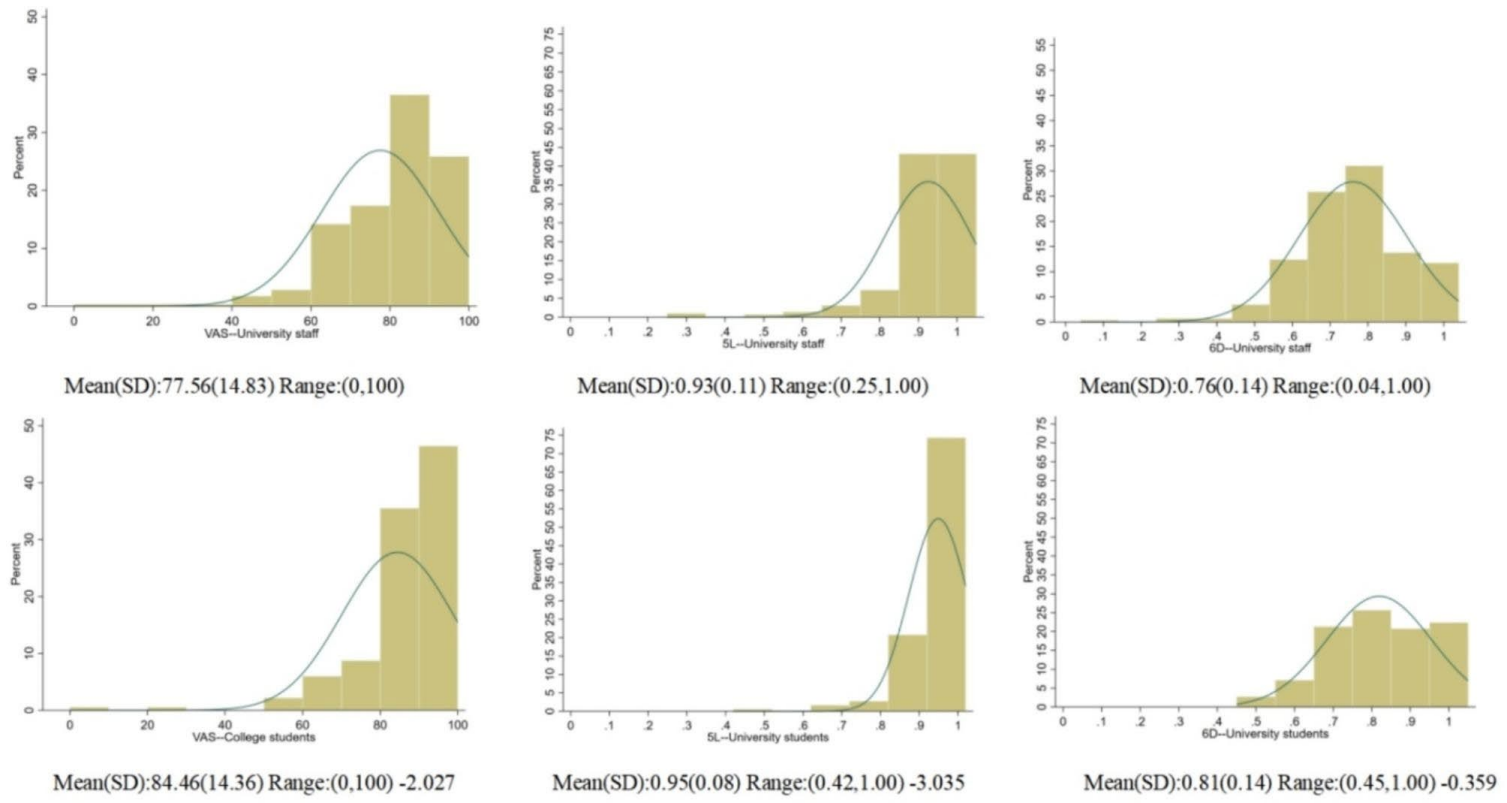
Distribution of EQ-VAS score,EQ-5D-5L,and SF-6DV2 utility scores in the two samples

For the students, the EQ-VAS and EQ-5D-5L HUS scores were severely skewed while the SF-6DV2 appeared to follow a uniform distribution. The skewness being -2.027, -3.035 and -0.359 for EQ-VAS, EQ-5D-5L and SF-6DV2 HUS all followed left-skewed distribution. SF-6DV2 HUS was more evenly distributed than the other two scores. While the HUS of EQ-5D-5L was more concentrated between 0.8 and 1.0; EQ-VAS was mainly concentrated on 80–100 (Fig.1).

### Agreement between the EQ-5D-5L and SF-6DV2 utility scores

**Fig. 2:**
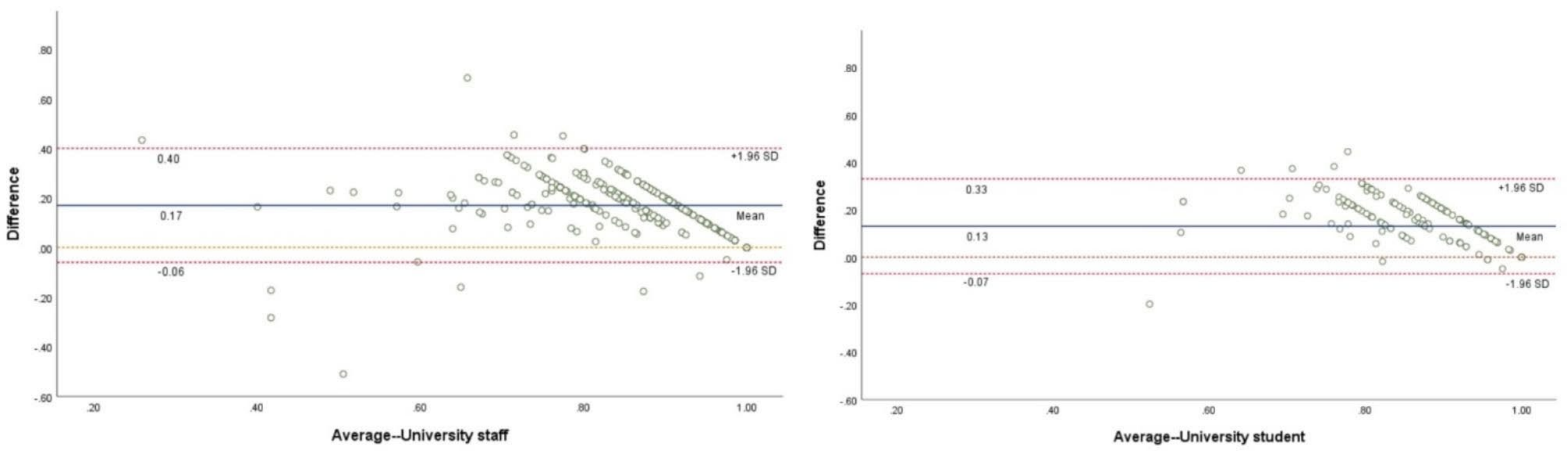
Bland-Altman plot of the EQ-5D-5L and SF-6DV2 utility scores in the two samples

The HUSs of two instruments were in moderate agreement with the ICCs being 0.567 and 0.553 for the staff and the students respectively. The agreement displayed by the Bland-Altman appeared to confirm this. The two samples Bland-Altman analysis all showed that over 95% points were within the limits of agreements (University staff: 99.95%; (University student: 99.97%). The HUSs by EQ-5D-5L were normally higher than those measured by the SF-6DV2. But in cases where subjects had low HUSs (< 0.6), EQ-5D-5L produced lower HUS than SF-6DV2. This observation appeared in both samples (Fig.2).

### Construct validity

According to Table5, several similar dimensions of EQ-5D-5L and SF-6DV2 failed to show good convergent as theoretically expected. Specifically, SF-6DV2 PF correlated weakly with EQ-5D-5L MO and SC dimensions in both samples. However, the EQ-5D-5L PD and AD dimensions showed strong correlations with the similar dimensions of the SF-6DV2 PN and MH, respectively. The correlation coefficients were 0.748 and 0.563 among staff; and 0.623 and 0.645 among students. Discriminant validity was suggested that SF-6DV2 MH dimension was not significantly correlated with the pure physical constructs, MO, SC or UA, of EQ-5D. What was noteworthy was that SF-6DV2 RL and VT constructs tended to had stronger and more significant correlations with PD and AD dimensions of EQ-5D-5L, rather than MO, SC or UA. This followed the previous report that SF-6DV2 is more socially oriented whereas EQ-5D-5L is more physically oriented.

**Table 5:** Correlations of the dimensions of EQ-5D-5L and SF-6DV2 in the two samples

SF-6DV2	EQ-5D-5L University staff					EQ-5D-5L University students				
	MO a	SC b	UA c	PD d	AD e	MO a	SC b	UA c	PD d	AD e
PF f	0.263**	0.223**	0.250**	0.418**	0.325**	0.238**	0.155*	0.210**	0.352**	0.300**
RL g	0.091	0.01	0.074	0.432**	0.453**	0.188*	0.027	0.091	0.371**	0.445**
SF h	0.146*	0.110	0.166**	0.428**	0.467**	0.174*	0.055	0.133	0.179*	0.447**
PN i	0.192**	0.136*	0.202**	0.748**	0.3861**	0.211**	0.047	0.120	0.623**	0.517**
MH j	0.02	0.009	0.022	0.426**	0.563**	0.141	0.001	0.121	0.412**	0.645**
VT k	0.107	0.129*	0.150*	0.487**	0.463**	0.095	0.046	0.130	0.475**	0.642**

**P < 0.01, *P < 0.05, a Mobility (MO); b Self-care (SC); c Usual activities (UA); d Pain/Discomfort (PD);

e Anxiety/Depression (AD); f Physical functioning (PF); g Role limitation (RL); h Social functioning (SF); i Pain (PN); j Mental health (MH); k Vitality (VT)

Correlations between EQ-VAS score, EQ-5D-5L and SF-6DV2 HUSs are shown in Table6. For university teachers, the coefficients were 0.592 (EQ-VAS and EQ-5D-5L HUS), 0.570 (EQ-VAS and SF-6DV2 HUS), and 0.692 (EQ-5D-5L and SF-6DV2 HUSs), respectively, all indicating a strong correlation. For the students, the coefficients were 0.421 (EQ-VAS and EQ-5D-5L HUS), 0.442 (EQ-VAS and SF-6DV2 HUS), and 0.703 (EQ-5D-5L and SF-6DV2 HUSs), respectively. Among them, EQ-5D-5L and SF-6DV2 HUSs have stronger correlation in both samples.

**Table 6:** Correlation of EQ-VAS score, EQ-5D-5L and SF-6DV2 utility scores in the two samples

	University staff			University students		
	EQ-VAS	EQ-5D-5L	SF-6DV2	EQ-VAS	EQ-5D-5L	SF-6DV2
EQ-VAS	1.000	0.592**	0.570**	1.000	0.421**	0.442**
EQ-5D-5L		1.000	0.692**		1.000	0.703**
SF-6DV2			1.000			1.000

**P < 0.001

### Known-groups validity and sensitivity of the utility scores

The results of known-groups validity and sensitivity for EQ-VAS score, EQ-5D-5L and SF-6DV2 HUSs are shown in Table7. Among university staff, EQ-VAS score, EQ-5D-5L and SF-6DV2 HUSs all found significant differences for two known-groups (with and without disease, with and without symptom or discomfort, and number of any of the three conditions). SF-6DV2 HUS was more efficient than EQ-5D-5L HUS and EQ-VAS score in detecting the three conditions (RE > 1 for both). On the other hand, EQ-5D-5L HUS and EQ-VAS score were more sensitive than SF-6DV2 HUS in identifying the staff with and without injury. EQ-5D-5L HUS was also more discriminative than EQ-VAS score for two known-groups (i.e., with and without symptom or discomfort, and with and without injury). In contrast, EQ-VAS score was more discriminative than EQ-5D-5L HUS in the two known-groups (with and without disease, and number of any of the three conditions).

**Table 7:** Known-groups validity and sensitivity of EQ-VAS score, EQ-5D-5L and SF-6DV2 utility scores in the two samples

Subject characteristics	n	University staff, Mean (SD)				n	University students, Mean (SD)		
		EQ-VAS	EQ-5D-5L	SF-6DV2			EQ-VAS	EQ-5D-5L	SF-6DV2
Disease a									
No	168	81.93(13.09)	0.95(0.09)	0.80(0.14)	159		84.22(15.00)	0.96(0.07)	0.83(0.14)
Yes	122	71.69(15.07)	0.89(0.12)	0.70(0.13)	24		86.15(9.03)	0.91(0.07)	0.75(0.09)
F statistics		36.942	24.515	40.426			0.605	2.999	2.611
p-value		< 0.001	< 0.001	< 0.001			0.546	0.003	0.010
RE		1.51	1.00	1.65			0.20	1.00	3.33
Symptom or discomfort in 12 months b									
No	134	83.20(10.84)	0.97(0.08)	0.84(0.12)	114		86.81(15.32)	0.98(0.03)	0.87(0.12)
Yes	156	72.90(16.06)	0.88(0.12)	0.69(0.13)	69		80.60(11.74)	0.90(0.10)	0.74(0.12)
F statistics		37.976	48.779	117.522			8.327	65.576	46.846
p-value		< 0.001	< 0.001	< 0.001			0.004	< 0.001	< 0.001
RE		0.82	1.00	2.41			0.12	1.00	0.71
Injury in 12 months c									
No	251	77.90(14.81)	0.93(0.11)	0.77(0.15)	129		85.12(13.23)	0.96(0.69)	0.84(0.13)
Yes	33	73.70(15.33)	0.89(0.13)	0.73(0.13)	54		82.48(16.80)	0.93(0.09)	0.79(0.13)
F statistics		1.528	3.37	1.268			0.516	7.457	9.623
p-value		0.128	0.068	0.139			0.348	0.001	< 0.001
RE		0.45	1.00	0.38			0.06	1.00	1.29
Three of the above									
0	98	84.18(10.81)	0.98(0.06)	0.85(0.12)	85		87.64(13.80)	0.98(0.03)	0.88(0.12)
1	84	78.46(14.51)	0.93(0.11)	0.76(0.13)	58		81.05(15.59)	0.94(0.09)	0.79(0.12)
2	86	71.65(15.63)	0.88(0.14)	0.68(0.13)	31		81.23(13.28)	0.90(0.08)	0.73(0.13)
3	16	63.44(12.74)	0.86(0.08)	0.67(0.07)	9		87.78(7.55)	0.87(0.10)	0.72(0.11)
F statistics		18.112	16.596	33.651			3.276	17.615	16.176
p-value		< 0.001	< 0.001	< 0.001			0.022	< 0.001	< 0.001
RE		1.09	1.00	2.03			0.19	1.00	0.92

a Disease: hypertension, diabetes, kidney disease, heart disease, allergic diseases, other diseases

b Symptom and discomfort: headache, back pain, pain in other areas; indigestion, nausea, sleep disturbance, emotional problems, and other discomforts

c Injury: Trauma (fracture, dislocated, muscle strain, cuts, abrasions, etc.), burns and scalds, and other conditions

Among the students, both EQ-5D-5L and SF-6DV2 HUSs could detect significant difference in all the known-groups. And EQ-5D-5L HUS was found to be better efficient than SF-6DV2 and EQ-VAS in detecting differences in two known-groups (with and without symptom or discomfort, and number of any of the three conditions) (RE < 1), while SF-6DV2 HUS was better efficient than EQ-5D-5L HUS in detecting disease and injury (RE > 1 for both).

## Discussion

Measurement performance of GPBMs varied a great deal across populations and GPBM instruments were normally not interchangeable. This phenomenon has necessitated the research on the psychometric performance of even widely-used GPBM in specific populations and decision-making settings. This study investigated the psychometric properties of EQ-5D-5L and SF-6DV2 of two samples populations living a life in the higher-education sector. The results showed that the EQ-5D-5L and SF-6DV2 HUSs had acceptable convergent validity and known-groups validity. Nevertheless, only EQ-5D-5L (PD and AD) and SF-6DV2 (PN and MH) showed the expected good convergent validity. Although HUSs of two questionnaires were in moderate agreement, they were not be interchangeable. The SF-6DV2 seems to be preferred in the study populations as it displayed a lower ceiling effect and better distributional property than the EQ-5D-5L.

Similar to the previous findings [17, 40], the EQ-5D-5L systematically yielded higher HUS than SF-6DV2 in both staff and students The HUS differences of 0.17 and 0.13 respectively for staff and students reached the statistical significances. This may suggest that EQ-5D-5L has overestimated the health status given its ceiling effect 5.7 and 2.54 times that of SF-6DV2. It further illustrates an important issue that the choice of HRQOL measurement tool would substantially affect the decision-making about resource allocation in the context of higher education. Two reasons could account for the differences. First, the EQ-5D-5L utility is determined by the self-ranked health status on the day of survey while the SF-6DV2 covers a longer period of health status over the past four weeks. Thus, the SF-6DV2 theoretically has captured more health-related problems than the EQ-5D [22]. For example, a respondent could be free of pain/discomfort on a single day but may have experienced it some time in the past four weeks. Second, the SF-6DV2 HUS has unique contribution for the dimension Vitality, which would reflect extra HRQOL impairment.

The overall agreement of the HUSs was moderate between the two instruments with ICC being 0.567 and 0.553. The value is lower than the ICC discovered in a sample (n = 19,177) drawn from the general population (ICC = 0.75) [15]. The visual inspection of agreement by Bland-Altman plots (Fig.2) demonstrated not only the systematically higher utility of EQ-5D-5L relative to SF-6DV2, but also some consistency. These findings are in line with prior results [41, 42]. The plots showed that, in the lower range of HUS, HUS differences increased, and interestingly, the EQ-5D-5L produced lower HUS than the SF-6DV2 when the HUS below certain threshold. This could be attributed to the difference in the utility scoring functions of the two instruments. That is, the difference in coefficients of the two functions is in general increased along the health severity; and the EQ-5D-5L scoring function tends to generate lower HUS for health states with severe or very severe problems (Table1).

Regarding the construct validity, strong correlations were observed between the similar dimensions (PD and PN; AD and MH) of EQ-5D-5L and SF-6DV2. However, the correlations were not as strong for other theoretically similar dimensions (MO/SC with PF in both samples, UA and SF among university staff). The finding in line with the results of a general population study in China [20], which may also be attributed to the different connotations of the similar dimensions. For instance, EQ-5D-5L MO/SC both involve simple activities (walking, bathing or dressing), whereas SF-6DV2 PF includes both high-intensity and moderate-intensity activities (running, lifting a table, etc.). In addition, the EQ-5D-5L puts emphasis on physical functions, while SF-6DV2 is more socially related [5, 43, 44]. In reality, usual activities can be performed without social contacts. So the UA dimension of EQ-5D-5L may not be strongly correlated with the SF dimension of SF-6DV2 in our case. In addition, we find that correlations seem to occur more frequently in the employee sample than in the student sample.There may be two possible reasons. Firstly, university staff have worse health than the students, hence the two instruments tend to converge in identifying the same HRQOL problems. Secondly, university students are likely to have a greater variety of daily activities than staff, and therefore PF correlates slightly less with MO and SC in university students than in university staff. The staff-student difference was supposed to be related to both occupations and age. So far the evidence is rare directly comparing the HRQOL of university staff and students. However, studies have shown that the younger age is associate with better health status [45, 46]. On the other side, there exists research indicating the suboptimal HRQOL of college teachers from an occupational health perspective [23]. We also found strong correlations among the three overall health indicators, showing convergent validity of two instruments in our study population. Furthermore, the degree of correlation between the HUSs was stronger than their correlations with EQ-VAS score. This may be due to the fact that both the two HUSs reflect health preferences of general Chinese population. This is different from the result of Thai general population that the correlation between EQ-5D-5L HUS and EQ-VAS score was stronger than their correlations with SF-6DV2 HUS [17]. The study utilized the SF-6D value set in the UK and the EQ-5D value set in Thailand, which may explain the differences in results.

In terms of known-groups validity, the HUSs and EQ-VAS scores have discriminated the majority of groups with known difference in health states, supporting that discriminant validity of the questionnaires. However, the exception occurred to the injury condition, for which both HUSs and EQ-VAS appeared weak to discriminate staff with or without injury. This finding may be attributed to two factors. Firstly, co-existence of disease and symptom/discomfort on the staff without injury in the last 12 months (75 university staff). Secondly, injury of university staff being minor. Meanwhile, they had different sensitivity in distinguishing the difference in HRQOL between the known-groups. The EQ-5D-5L and SF-6DV2 HUSs are generally better than the EQ-VAS score. One potential reason is that the two HUSs are based on the information on the five or six health aspects while the EQ-VAS score reflects the global health of an individual which is insensitive to health impairment in a certain dimension. This is similar to the finding in depressed patients that SF-6D HUS had better sensitivity over EQ-VAS score [47]. With regard to the sensitivity of the two HUSs, we found that either of them is not superior to the other. Previous studies also reported inconsistent findings in general populations in Asia [17, 48]. Apart from the differences in study design, population, method (e.g., the order of two instruments), the finding could also be due to their scoring functions: the EQ-5D-5L function apts to yield lower HUS for severe health states offsetting the advantage in descriptive system of SF-6D (more dimensions).

The strength of our study is the randomized assignment of the two instruments thus avoiding the order effect [49]. Our study also has two limitations. First, it is a cross-sectional study thus the test-retest reliability and responsiveness cannot be assessed. Second, HRQOL were collected by participants completing the paper version of instruments online. This practice might have affected the quality of data. Nevertheless, the participants are highly-educated and familiar with internet use, which would ensure the validity and reliability of HRQOL to a large extent.

## Conclusion

In conclusion, it appears that both EQ-5D-5L and SF-6DV2 HUSs have acceptable measurement properties including convergent validity, known-groups validity, sensitivity in Chinese university staff and students. However, only EQ-5D-5L (PD and AD) and SF-6DV2 (PN and MH) demonstrated the anticipated good convergent validity. Future studies are warranted to further evaluate other measurement properties such as test-retest reliability and responsiveness of the two instruments in the populations.

### Electronic supplementary material

Below is the link to the electronic supplementary material.

Supplementary Material 1

## Data Availability

Data is not suitable for public deposition due to ethical concerns. Requests for data may be sent to the corresponding author: Wang Pei; Email Address: wang_p@fudan.edu.cn.
