# Supplementary material for: Psychometric performance of EQ-5D-5L and SF-6DV2 in measuring health status of populations in Chinese university staff and students
Source: BMC Public Health. 2023 Nov 22;23:2314. doi: 10.1186/s12889-023-17208-z (PMC10664374; doi:10.1186/s12889-023-17208-z)
Supplement: Supplementary file 1 — Supplementary Material 1 [file 12889_2023_17208_MOESM1_ESM.doc]

Appendix 1 EQ-5D and SF-6D dimensions according to their similarities

|  | EQ-5D | SF-6D |
| --- | --- | --- |
| Dimensions | Mobility | Physical function |
|  | Selfcare |  |
|  | Usual activity | Role limitation |
|  |  | Social function |
| 、 | Pain/Discomfort | Pain |
|  | Anxiety / Depression | Mental health |
|  |  | Vitality |
